# Human Health Risks from Low-Level Environmental Exposures: No Apparent Safety Thresholds

**DOI:** 10.1371/journal.pmed.0020350

**Published:** 2005-10-18

**Authors:** Donald T Wigle, Bruce P Lanphear

## Abstract

Wigle and Lanphear argue that for many toxins widely dispersed in the environment, even very low levels pose health risks.

Recent developments in environmental epidemiology offer the promise of strengthening human health protection. Regulatory agencies responsible for protecting human health from environmental hazards assess data on relationships between exposure levels and adverse health effects to develop limits for contaminant levels in air, water, food, soil, house dust, and consumer products. Most regulatory agencies assume that there is no safe level of exposure to carcinogens and use linear dose-response models to estimate human health risks at low exposure levels. In contrast, regulators usually assume that a threshold, or “safe,” exposure level exists for noncarcinogens.

## Risk Assessment

In conducting risk assessments to characterize potential adverse health effects of human exposures to environmental hazards [1], regulators depend on experimental animal studies in the absence of adequate epidemiologic data. These studies are critical to uncover health effects before human exposure occurs (e.g., premarket testing of a new chemical) whereas epidemiologic studies can be used to directly evaluate health effects among exposed persons. The difficulty of directly measuring health risks at very low exposure levels can be an important limitation of both epidemiologic and toxicologic studies.

Sources of uncertainty in conventional animal studies include: (1) the much shorter exposure period compared to humans, (2) testing is often limited to adult (but not pregnant, newborn, or sexually immature) animals, (3) use of genetically homogeneous animals (with loss of the ability to detect potentially heightened risks among genetically diverse subgroups, such as exist in human populations), (4) the use of very high doses of test chemicals (e.g., administration of high doses of a teratogenic toxicant to pregnant animals may cause early pregnancy loss before birth defects can be readily observed), (5) small numbers of test animals, and (6) the need to extrapolate across species to humans [2].

For instance, neurotoxic effects of prenatal or early-life exposure to lead, polychlorinated biphenyls, and methylmercury in humans occur at intake levels about three orders of magnitude lower than those predicted from rodent data [3]. The role of potential biases and crude exposure indices in producing uncertainties in epidemiologic studies has been reduced, to some degree, by the increasing use of improved study methodologies, e.g., the use of exposure and susceptibility biomarkers.

There are higher risks per unit exposure dose at low exposure levels.

The following case studies for four of the most widespread and extensively studied environmental hazards show that (1) there is no apparent threshold for health risks with dose-response relationships over exposure ranges far below those generally used in animal studies, and, in some cases, (2) there are higher risks per unit of exposure dose at low exposure levels.

## Case Studies

### Lead

Lead is a potent neurotoxin capable of causing severe childhood brain damage at blood lead levels only 2- to 3-fold higher than those that cause no overt symptoms. Overt lead poisoning has been recognized for centuries, but there was no convincing evidence of IQ deficits at relatively low-level lead exposure until 1979 [4]. Noting the lack of a lead exposure threshold for impaired cognitive function and heme synthesis, the Environmental Protection Agency has not specified a safe exposure level. The United States Centers for Disease Control and Prevention do not recommend public health or medical actions for children unless their blood lead level exceeds 0.48 μM (10 μg/dl), a level about 100-fold higher than that estimated for pre–Industrial-Age children [5]. Epidemiologic studies of children in several countries found inverse relationships between IQ and blood lead levels over a range extending below 0.48 μM, with no evidence of a threshold [6].

In a recent study of over 4,000 children, scores on four cognitive test subscales (math, reading, block design, digit span) were inversely associated with current blood lead levels, even in analyses restricted to those with blood lead levels less than 0.48 μM (<10 μg/dl) [7]. The inverse relationship for math and reading scores persisted in the subgroup with blood lead levels less than 0.24 μM (<5 μg/dl), and reading deficits per unit blood lead increment were greater among those with lower blood lead levels.

Similarly, another birth cohort study found an inverse relationship between IQ at age 10 to 12 years and quartiles of tibial bone lead concentration with the greatest IQ decrement occurring between the lower two quartiles [8]. Two longitudinal US birth cohort studies found inverse relationships between full-scale IQ and blood lead among children whose blood lead level since birth never exceeded 0.48 μM [9,10]. In a pooled analysis of seven of the eight prospective longitudinal studies, the investigators reported that the average IQ deficit associated with an increase in concurrent blood lead concentration from less than 0.048 μM to 0.48 μM was about 3-fold higher than the average IQ deficit associated with an increase in concurrent blood lead concentration from 0.48 μM to 0.96 μM [11]. The latter report included a log-linear model for IQ versus concurrent blood lead concentration, including adjustment for HOME Score (Home Observation for Measurement of the Environment, a standardized measure of the home environment), maternal education, maternal IQ, and birth weight, that clearly showed the steeper dose-response relationship at low blood lead levels (Figure 1).

**Figure 1:**
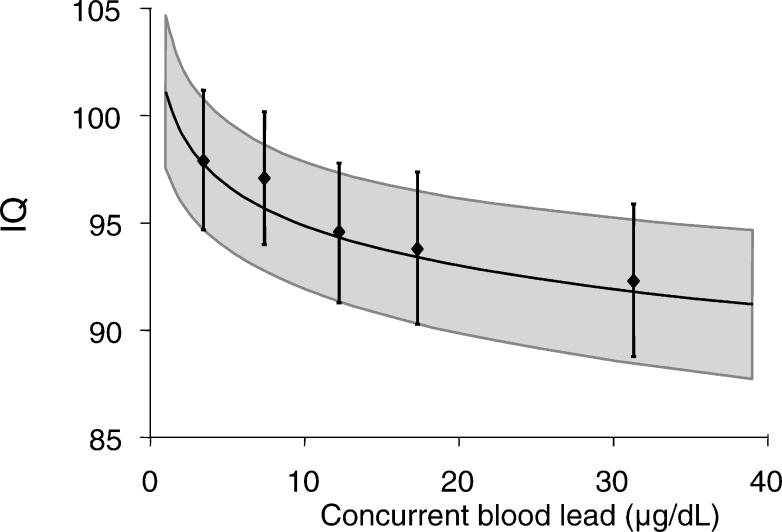
Log-Linear Model for IQ Versus Concurrent Blood Lead Concentration, Adjusted for HOME Score, Maternal Education, Maternal IQ, and Birth Weight The mean IQ (95% confidence intervals) for the intervals <5 µ/dl, 5–10 µ/dl, 10–15 µ/dl, 15–20 µ/dl, and >20 µ/dl are shown. (Figure by authors, adapted from [11]).

### Tobacco smoke

There is convincing epidemiologic evidence that prenatal maternal active smoking impairs fetal growth. A US prospective study demonstrated an inverse nonlinear relationship between term birth weight and third-trimester smoking intensity, with larger birth weight decrements at low maternal smoking intensities [12].

It now appears that even low-level exposure to environmental tobacco smoke (ETS), or “passive smoking,” can reduce fetal growth. In a Finnish study of nonsmoking women, the risk of preterm birth was dose-related to self-reported prenatal maternal ETS exposure intensity and maternal hair nicotine levels [13]. Glutathione-S-transferase (GST) enzymes detoxify many chemicals, including polycyclic aromatic hydrocarbons and certain other toxicants present in tobacco smoke. A Korean study of nonsmoking women found that combined maternal ETS exposure and null polymorphisms of two GST genes involved in tobacco smoke metabolism (GSTT1 and GSTM1) were associated with birth weight deficits [14]. A US study of over 4,000 children age 6 to16 years found inverse dose-response relationships between serum cotinine (the major metabolite of nicotine) and scores on reading, math, and visuospatial reasoning independent of several potential confounders [15]. Importantly, the dose-response relationship between reading scores and serum cotinine was stronger among children with cotinine concentrations below 0.5 ng/ml compared to those with higher levels.

### Radon

An expert committee recently concluded that the most plausible relationships between low-level ionizing radiation and mutations, chromosome aberrations, and cancer are linear, with no threshold [16]. The high radon levels in the air of some underground mines cause lung cancer among occupationally exposed men, the risk being a linear function of cumulative radiation dose [17]. A pooled analysis of eight epidemiologic studies of underground miners showed that the excess risk of lung cancer per unit of cumulative radon exposure was greater at lower exposure levels [18]. Among men with the same cumulative radon exposure, therefore, prolonged exposure at low levels is more hazardous than shorter exposures at higher levels.

Indoor air radon levels vary widely in homes and other buildings. Average cumulative radon doses from lifetime residential exposures are about 10-fold lower than those among exposed miners. Despite the relatively low average radon levels in homes, combined analysis of 17 epidemiologic studies showed that persons with time-weighted average residential radon exposures of 150 Bq/m3 (the current level above which the Environmental Protection Agency recommends actions to confirm radon levels and sources and the need for remedial measures such as ventilation) had a 24% (95% CI 11%–38%) increased lung cancer risk [19]. Thus, directly measured lung cancer risk at relatively low radon levels in the general population is consistent with an estimate based on linear extrapolations of risks for miners with much higher average exposures [20].

### Chlorination disinfection by-products in drinking water

During disinfection of drinking water, chlorine reacts with naturally occurring organic material and produces many by-products of disinfection, including the trihalomethanes (THMs) chloroform, bromodichloromethane, dibromochloromethane, and bromoform, that are known animal carcinogens. Based on a risk assessment of kidney tumors in rats chronically exposed to high chloroform doses, Health Canada concluded that the human lifetime cancer risk associated with drinking water containing THMs at 100 μg/l (the current Canadian THM drinking water guideline) would be negligible [21]. However, a recent pooled analysis of six epidemiologic studies of human bladder cancer with over 8,000 subjects showed that men exposed to THM levels above 1 μg/l had a 24% increased bladder cancer risk compared to less exposed men, representing an excess lifetime bladder cancer risk of about seven per 1,000 [22]. This risk is much higher than those usually designated as negligible (regulatory agencies have variably defined negligible risk as a lifetime excess risk of 10^−6^ to 10^−5^). Thus, a risk assessment of THMs based on carcinogenicity of chloroform in animals may greatly underestimate human cancer risk.

## Conclusion

In contrast with animal studies, epidemiologic studies can be used to assess health risks at exposure levels prevalent in human populations. Findings from some of the most thoroughly studied and widely dispersed environmental contaminants indicate that there is no apparent safe exposure level. Indeed, in some cases, there are greater risks for a given exposure at the relatively low exposure levels most prevalent in human populations. Environmental chemicals should be thoroughly evaluated for toxicity before they are marketed [23], but when available, epidemiologic data should preferentially be used to develop environmental standards and to assess the adequacy of existing standards based on experimental animal studies.

The public depends on decision makers, scientists, and regulators to restrict exposure to widespread toxins that have known or suspected serious potential health effects. We hold that risk assessments should not assume thresholds for noncarcinogens as well as carcinogens, especially for toxins shown in epidemiologic data to exhibit no apparent threshold and those not yet adequately tested for developmental toxicity. The four major toxins reviewed here are widely dispersed in the environment and emerging evidence indicates that exposures must be virtually eliminated to protect human health. It would be imprudent to assume that there are not other widely distributed environmental toxins or chemicals with the potential to cause adverse human health effects at exposure levels currently considered to be “low.”

## Abbreviations

ETS: environmental tobacco smoke
GSTM1: glutathione S-transferase M1 gene
GSTT1: glutathione S-transferase T1 gene
THM: trihalomethane
